# Books Reviewed

**Published:** 1869-04

**Authors:** 


### Books Reviewed.

Treatise upon Diseases of the Ear, including the Anatomy of the Organ. By Anton von Tröltsch, M. D. Translated and edited by D. B. St. John Roosa, M. D. Second American from the fourth German edition. New York: Wm. Wood & Co., 1869.

A text-book which embraces the whole field of aural medicine and surgery, and which is based upon careful observation and thorough investigation, is certainly to be received by the profession with great favor. It would, at first, seem impossible that a volume of near six hundred pages could be written upon the ear, without supplying deficiencies of knowledge with fancy sketches of disease; but when we remember that diseases of the ear are now receiving the attention which their importance demands, and that otology is taking rank with ophthalmology and other branches of special surgery which have recently been enriched by the discovery of so much truth as to have already become new sciences, our wonder subsides and we are ready to presume that the work is really based upon established fact.

We have carefully examined this work upon the ear, with the view of discovering, if possible, if otology had actually reached to a completeness which would require so extensive a text-book to embrace it, and we find that though the whole present science is presented with great completeness, still nothing too much has been said; the author and editor both anticipate the rapid growth of knowledge in this department, believing that only the half of what is to be known is yet discovered.

The work is a plain, practical and comprehensive treatise upon the diseases of the ear. The subjects are all treated with great care, and illustrated with cuts wherever necessary to correct understanding; many of the illustrations are exceedingly useful in conveying correct idea of parts and their relations and of the modes of instituting measures of relief and cure.

The thanks of the profession are due Dr. Roosa both for the translation and for valuable contributions to the text. As now presented the work must stand as *the* text-book in otology both for schools of medicine and practitioners.

A hand-book of Uterine Therapeutics and of Diseases of Women. By Edward John Tilt, M. D. Second American edition, thoroughly revised and amended. New York: D. Appleton & Co., 1869.

Uterine therapeutics at the present time in the history of medicine "draws" largely upon the thoughts and efforts of the profession, and as this work has been thoroughly revised and amended, it must receive all due attention. In its first edition it was one of the most suggestive and instructive works upon the treatment of uterine diseases, and certainly in revised and amended form it is an attraction.

It embodies a thorough discussion of all the remedial measures proposed for the cure of these diseases, and also contains an account of important uterine diseases and their modes of cure, which would do credit to the most pretentious works upon Diseases of Women. The work is characterized by a common sense view of diseases and their modes of cure, which gives it much of its deserved popularity with the working portion of the profession.

Uterine pathology is the growth of a quite recent date. Racamier, in 1816, showed the uses of the speculum, and its necessity in the diagnosis and treatment of uterine diseases; previous to that time the most irrational and absurd views prevailed. The use of this instrument and the application of local remedies to the relief of uterine diseases has now become universal, but it is within our own distinct recollection when almost all our present modes of treatment were opposed and ridiculed by some wise-acres in the profession with bitterness worthy a better cause. The present modes of treating uterine diseases are well described in the work before us, and we earnestly commend it to careful perusal.

---

Accidental and Congenital Atresia of the Vagina, with a Mode of Operating for successfully establishing the Canal. By Thomas Addis Emmet, M. D.

This paper was read before the New York Obstetrical Society and published in the *Richmond Medical Journal.* The author speaks first of the strong tendencies to contraction after dividing with a knife in case of occlusion or absence of vagina, so that in all, or nearly all cases, the artificial vagina becomes obliterated or narrowed down to a mere fistulous tract. To obviate this, he proposes to divide the tissues with the scissors or by laceration as far as possible with the fingers. When thus divided or lacerated, the parts have not according to his experience the same tendency to contract during the process of healing as when separated by a clean cut. He illustrates these facts with detail of cases. The paper is a highly interesting one and the subject worthy of attention.

---

A New Operation for Artificial Anchylosis, illustrated by two cases. By Lewis A. Sayer, M. D.

This pamphlet is a republication from the Transactions of the State Medical Society of 1846, of a paper on "A New Operation for Artificial Hip-joint in Bony Anchylosis." The object, the author informs us, is to vindicate scientific truth and his own reputation from the "false" statements of Dr. Louis Bauer in his recent work published by Wm. Wood & Co. The case is re-published and letters from various gentlemen who saw the case, added. We have no disposition to express any opinion as to the merits of the controversy, but certainly Dr. Sayer seems to have fully proved by competent witnesses the accuracy of his report. If physicians are at all to be believed, Dr. Sayer's cases fully sustained it.

Structural Lesions of the Skin; their Pathology and Treatment. By Howard F. Damond, M. D. Philadelphia: J. B. Lippincott & Co., 1869.

The author informs us that "the structural lesions of the skin consist in hypertrophy, atrophy, and pathological new formations," and under these general heads he arranges his consideration of a great number of the rare and some of the common structural changes of the skin. He has chosen congenital canities, cutaneous horns of forehead, elephantiasis arabum, and cutaneous horn of eyelid, as subjects for illustration, and the cuts showing these forms of disease are very beautiful.

Canities, or hair without color, he says, is rarely congenital, has been noticed in a few cases only on one side. A few well authenticated cases are recorded of sudden withdrawal of the coloring principle of the hair, or some chemical change has destroyed the pigment. We have *heard* of hair turning white in a single night from sudden fright, but have not supposed the accounts well authenticated. The cases of elephantiasis are finely illustrated, and the illustration of the horn of the eyelid is quite remarkable. The work is prepared in very attractive style, and the rare and less known structural changes of the skin are very thoroughly and attractively presented. It is our only exclusive treatise upon these obscure forms of disease, so far as we are informed, and all who take interest in dermatology and the rare and important forms of skin diseases here presented will be deeply interested in the work.

---

A History of the Medical Department of the University of Pennsylvania, with sketches of the lives of deceased Professors. By Joseph Carson, M. D. Philadelphia: Lindsay & Blakiston, 1869.

This work, by Dr. Carson, is a very pleasant contribution not only to the history of the medical department of the University of Pennsylvania, but of the profession generally and of medical teaching in this country. The biographical sketches are of those of the highest reputation in the profession, and will be read with deep interest by many. The work contains many facts in the history of medicine which all will be interested in reading, but especially will the Alumni of the University of Pennsylvania be gratified by the publication of this volume.

---

Pennsylvania Hospital Reports. Vol. ii, 1869. Lindsay & Blakiston.

This is the second volume of the Hospital Reports, the first appearing about one year since. It consists mainly of papers based on observations made at the hospital, which are consequently eminently practical in character. To the hospital observation is often added the general experience of members of the staff, thus making all the papers exceedingly valuable.

Those physicians who are in possession of the first volume, or who have become in any way acquainted with the value of the work, will be much gratified with the announcement of the appearance of the second volume. It is one of the most attractive books of the year for physicians, and fully establishes the necessity of the yearly appearance of a similar volume. If space would permit we should give our readers an account of the important papers it contains, but to do so is impossible. All must accept the assurance of its value, and if possible, satisfy themselves by personal examination. It is edited by J. M. Da Costa and William Hunt, who have also written many of the most valuable papers which the book contains. We most earnestly hope that each succeeding year will furnish the profession a similar volume of equal merit.

On the Microscope in the Diagnosis and Treatment of Sterility. By J. Marion Sims, M. D. New York: D. Appleton & Co., 1869.

The author prefaces by speaking of incising and otherwise dilating the os uteri, and refers to the fact of his being accused of cutting open the cervix uteri recklessly and unnecessarily. He says: "So far as incision of the cervix uteri for dysmenorrhœa in the abstract, without reference to the sterile state, I wish it to be understood that I have nothing to recant, nothing to undo. But, so far as this operation may be indicated in cases of sterility, properly speaking, I candidly confess that I have a word of advice for my younger brethren; for I am now convinced that I have repeatedly cut open the cervix uteri, for the sterile state, when the operation was both useless and unnecessary." He proposes to settle with the microscope three questions, viz.: if we have semen with spermatozoa, if spermatozoa enter the utero-cervical canal, and if the secretions of this canal are favorable or not to the vitality of the spermatozoa.

The whole paper must be read in order to gain any just idea of the author's teaching. The pamphlet can be obtained of the publishers and is worthy of perusal.

